# Non-curative treatment of patients with oral tongue squamous-cell carcinoma

**DOI:** 10.1007/s00405-019-05456-y

**Published:** 2019-05-08

**Authors:** R. Mroueh, A. Haapaniemi, T. Saarto, L. Grönholm, R. Grénman, T. Salo, A. A. Mäkitie

**Affiliations:** 1 Department of Otorhinolaryngology-Head and Neck Surgery, University of Helsinki and HUS Helsinki University Hospital, P.O. Box 263, 00029 HUS FI-00029, Finland; 2 Department of Oncology, University of Helsinki and HUS Helsinki University Hospital, Helsinki, Finland; 3 Department of Otorhinolaryngology-Head and Neck Surgery, University of Turku and Turku University Hospital, Turku, Finland; 4 Cancer and Translational Medicine Unit, University of Oulu, Oulu, Finland; 5 Medical Research Unit, Oulu University Hospital, Oulu, Finland; 6 Oral and Maxillofacial Diseases, University of Helsinki, Helsinki, Finland; 7 Haartman Institute, Helsinki, Finland; 8 Research Program in Systems Oncology, Faculty of Medicine, University of Helsinki, Helsinki, Finland; 9 Division of Ear, Nose and Throat Diseases, Department of Clinical Sciences, Intervention and Technology, Karolinska Institutet and Karolinska Hospital, Stockholm, Sweden

**Keywords:** Head and neck cancer, Tongue cancer, Palliative care, Palliation, Surgery, Chemotherapy, End of life, Quality of life, Death

## Abstract

**Purpose:**

Late-stage OTSCC is associated with poor overall survival (OS). Non-curative treatment approach aims to improve quality of life and prolong survival of patients deemed incurable. The purpose of this study was to investigate the used non-curative treatment modalities for OTSSC and patient survival.

**Methods:**

All patients diagnosed with OTSCC and treated with non-curative intent at the HUS Helsinki University Hospital (Helsinki, Finland) during the 12-year period of 2005–2016 were included. Survival analysis after the non-curative treatment decision was conducted using the Kaplan–Meier method in this population-based study.

**Results:**

Eighty-two patients were identified. A non-curative treatment decision was made at presentation without any previous treatment in 26 patients (7% of all patients diagnosed with OTSCC during the study period). Palliative radiotherapy was administered to 24% of all patients. The average survival time after the non-curative treatment decision was 3.7 months (median 2 and range 0–26).

**Conclusions:**

Due to the short mean survival time after decision for treatment with non-curative intent, and the notable symptom burden in this patient population, a prompt initiation of all non-curative measures is warranted.

## Introduction

Non-curative treatment constitutes an interdisciplinary treatment effort for patients whose disease is unresponsive to approaches with curative intent and involves life-prolonging treatment and palliative care. Some patients, either at presentation, during the course of therapy or later during follow-up, will experience advanced and progressive disease, which may require a shift in the objective of medical care. Although life-prolonging treatment, also called disease-modifying or disease-stabilising treatment, can provide prolonged disease control with the current armamentarium of treatment modalities, side effects often reduce the patient’s quality of life. Recently, more research has also been aimed at further developing palliative care. Development has also been made in symptom management of advanced disease, as the impact of various aspects of palliative care is better recognized [1–4].

End-stage head and neck cancer (HNC) typically has profound effects and symptom burden on the patient’s airway, upper gastrointestinal tract, major senses, physical appearance, and self-esteem as well as social life and normal daily functions. Palliative care modalities, which also include radiotherapy and surgical interventions, play a critical role in improving quality of life of the patient by alleviating tumour-related symptoms. Still, the functional deficits and symptomology caused by HNCs, due to the distinctive profile of the cancer itself in combination with the organs and tissues affected, remain a challenge even for experienced multidisciplinary centres [4, 5].

While early stage oral tongue squamous-cell carcinoma (OTSCC) has a favourable prognosis with high cure rates, late-stage OTSCC is associated with poor overall survival (OS). Despite improvements in the treatment outcomes of OTSCC in Finland, significant morbidity and mortality rates still affect this patient population, and a considerable proportion of patients will succumb to their disease. Furthermore, several patients each year present with end-stage disease and are unsuitable for curatively intended therapy [6]. There is a dearth of data on non-curative treatment related to oral cancer. Moreover, no previous study to our knowledge has specifically focused on life-prolonging treatment or palliative care of end-stage OTSCC patients. Such studies would provide insight on various clinical aspects of palliative care for OTSCC and allow improvement of treatment protocols. Therefore, the purpose of this study was to investigate the implemented non-curative, i.e., life-prolonging treatment and palliative care, modalities of OTSCC, and patient survival at the HUS Helsinki University Hospital during a 12-year period from 2005 to 2016.

## Patients and methods

All patients diagnosed with OTSCC and treated with non-curative intent at the HUS Helsinki University Hospital (Helsinki, Finland) during the 12-year period between January 1st, 2005 and December 31st, 2016 were included. Subjects were identified from hospital registries with the ICD-10 diagnosis codes (C02.0, C02.1, C02.2, C02.3, and C02.9). Only histologically verified cases of epithelial cancer located in the mobile tongue were included.

Clinicopathological data were obtained from hospital records for the following parameters: age, sex, smoking and drinking habits, previous cancers, dental status, date of pathological diagnosis, tumour histopathology, tumour location, TNM class, treatment of the primary tumour, reconstruction, neck dissection, radio- and chemotherapy, use of tracheostomy or percutaneous endoscopic gastrostomy, date of surgery, date of start and end of radiotherapy, disease recurrence (location and date of diagnosis), date of non-curative treatment decision, date of the beginning of non-curative treatment, date and status at last follow-up at the palliative unit, and date and place of death.

Non-curative treatment comprises life-prolonging treatment and palliative care. While life-prolonging treatment was administered with the intention to improve patient survival with means of chemo-, radio, or chemoradiotherapy, palliative care was considered to have been started after the termination of curatively intended or life-prolonging treatment. We use the term “palliative treatment” when referring to surgery or radiotherapy given with non-curative intent with the aim to alleviate symptoms, not to prolong life. Patient survival analysis after decision of treatment with non-curative intent was conducted using the Kaplan–Meier method with R (version 3.5.1). Clinical data on a subgroup of the patients diagnosed and treated during 2005–2009 have been published previously [6]. Research permission for the study design was granted by the National Institute for Health and Welfare (Dnro THL/264/5.05.00/2015).

## Results

**Fig. 1:**
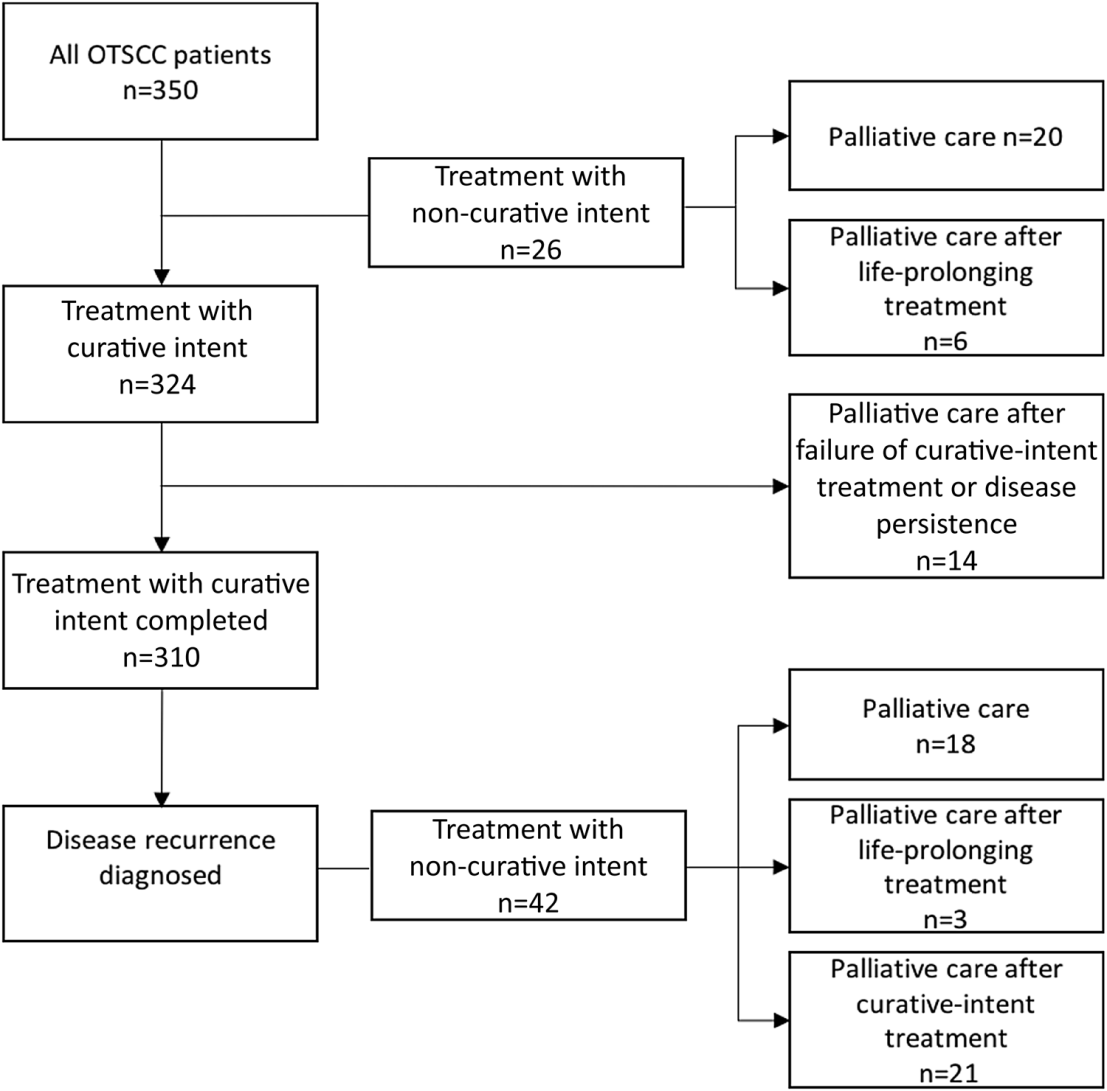
Consort flow diagram: OTSCC patient selection

Three hundred and fifty patients with primary OTSCC were identified (42% female) during the 12-year study period. A total of 82 (23%) out of the 350 patients received treatment with non-curative intent (40% female; mean age 66.1 years; median 65, range 28–94.4) at some phase of their management. A non-curative treatment decision was made at presentation without any previous treatment in 26 patients (7% of all patients diagnosed with OTSCC during the study period). Palliative care was the primary goal of treatment without any previous treatment for 20 patients (24% of the 82 patients who received treatment with non-curative intent and 6% of all 350 patients diagnosed with OTSCC during the study period). Palliative care was administered for 14 patients because of failure of curative treatment or persistent disease. Clinical characteristics of the study group are presented in Table 1 and the flowchart showing various subgroups in Fig. 1.

**Table 1:** Clinical characteristics of 82 OTSCC patients treated with non-curative intent in Helsinki during 2005–2016

Clinical characteristics	*n* (%)
Sex	
Male	49 (60)
Female	33 (40)
Age	
< 39	3 (4)
40–59	18 (22)
60–79	48 (59)
> 79	13 (16)
Stage of disease for patients treated only with non-curative intent (*n* = 26)	
I	1 (4)
II	3 (12)
III	1 (4)
IV	20 (77)
No data	1 (4)
Tobacco use, cigarettes per day	
Non-smoking	19 (23)
< 10	2 (2)
≥ 10	45 (55)
No data	16 (20)
Alcohol use, drinks per week	
> 24	29 (35)
Place of death	
Hospital	30 (37)
Hospice	16 (20)
Home	13 (16)
Health care centre	10 (12)
Nursing home	3 (4)
Unknown	10 (12)

Forty-six (56%) patients reported tobacco use and twenty-nine (35%) heavy alcohol consumption (defined in our study as more than 16 alcohol units for women and more than 24 alcohol units for men a week according to the Finnish national guidelines on classifying alcohol consumption) at the time of diagnosis. The majority of patients (*n* = 45, 54%) were found to be in need of dental treatment.

Of the patients receiving either only palliative care (*n* = 20, 24%) or life-prolonging treatment (*n* = 6) during the primary phase of treatment without any previous treatment, 20 (77%) had Stage IV disease, and of these, three were diagnosed with distant metastatic disease at presentation and three had already been diagnosed with another end-stage cancer. In five (6%) patients, all diagnosed with Stage IV disease, a palliative care plan was adopted after failure of curatively intended treatment. After surgical treatment, two of these patients received palliative radiotherapy and three received only symptomatic treatment. Disease control after primary phase treatment with curative intent, defined as no disease persistence or disease recurrence within 3 months after completion of treatment with curative intent, could not be accomplished in nine patients (2.6% of all the patients diagnosed and treated for OTSCC in our center during the study period), who were subsequently treated either with life-prolonging treatment (three patients) or only with palliative care (Fig. 1).

Forty-two patients (51% of patients treated with non-curative intent) received non-curative treatment after disease recurrence. Twenty-one (26%) patients experienced locoregional disease recurrence and distant metastases were diagnosed in 27 (33%) patients. It should be noted that, in one patient, the nature of a hypopharyngeal tumour could not be confirmed histologically as a recurrence and could have been a second primary tumour.

The average treatment delay after the non-curative treatment decision for patients receiving life-prolonging treatment was 19 days (median 19 and range 0–56). For patients treated with palliative surgery or radiotherapy, the average treatment delay after treatment decision was 25 days (median 18, range 5–167). Correspondingly, the mean interval time from diagnosis of primary or recurrent disease to treatment for patients administered life-prolonging treatment was 50 days (median 31 and range 0–266) and the mean interval time between start of life-prolonging treatment and shift to palliative care was 165 days (median 142, range 15–452).

Twenty (24%) patients received life-prolonging treatment. Chemotherapy, with or without combination radiotherapy, was used for 13 (16%) patients. Six patients received cetuximab in combination with 5-fluoroacil (5-FU) and cisplatin and four patients received paclitaxel with or without concomitant carboplatin. Boron neutron capture therapy (BNCT) was administered to six patients (7%). Invasive surgical procedures were undertaken in a non-curative setting in 6% (*n* = 5) of patients. As palliative treatment, one patient underwent salvage surgery, and two total glossectomies and one debulking neck surgery were performed. Palliative radiotherapy was administered to 20 (24%) patients in the form of conventionally fractionated radiotherapy with a total dose ranging from 6 to 30 Gy in fractions of 2 to 3 Gy. Almost half of all patients (48%; *n* = 39), and 31% (*n* = 8) of patients treated without any curative intent, received only symptomatic treatment without any adjuvant treatment. Percutaneous endoscopic gastrostomy (PEG) feeding was pursued in 53 (65%) patients; 23 of whom (28% of all patients) also underwent tracheostomy (Table 2).

**Table 2:** Treatment with non-curative intent given to 82 patients diagnosed with OTSCC in Helsinki during 2005–2016

Treatment	*n* (%)
Palliative surgery	3 (4)
Palliative surgery and radiotherapy	2 (2)
Palliative radiotherapy	18 (22)
Life-prolonging radiotherapy	7 (9)
Life-prolonging chemotherapy	7 (9)
Life-prolonging chemoradiotherapy	6 (7)
Only symptomatic treatment	39 (48)
	82 (100)
PEG feeding	30 (37)
Tracheostomy	2 (2)
PEG feeding and tracheostomy	23 (28)
No PEG or tracheostomy	27 (33)
	82 (100)

In the whole patient series, only one patient (female, age 94) refused the offered curatively intended treatment and received subsequently only symptomatic treatment. Among the patients treated with non-curative intent, one patient had declined the recommended neck dissection and one patient had declined radiotherapy as part of the initial curative treatment. In two cases, the ongoing palliative radiotherapy had to be interrupted due to lack of treatment compliance.

**Fig. 2:**
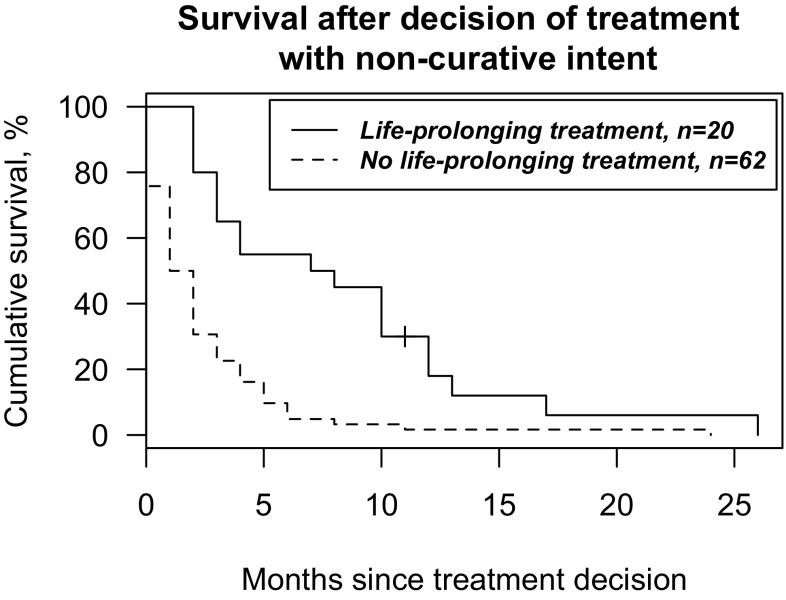
Overall survival after non-curative treatment decision with grouping according to treatment for 82 patients diagnosed with OTSCC at the Helsinki University Hospital during 2005–2016

The average survival time after the non-curative treatment decision was 3.7 months (median 2 and range 0–26). For patients administered life-prolonging treatment and for patients who received only palliative care without previous life-prolonging treatment, the average survival times after decision of treatment with non-curative intent were 7.9 (median 7, range 2–26) and 2.5 months (median 1.5, range 0–24), respectively. For patients treated with palliative surgery and/or radiotherapy and for patients receiving only symptomatic treatment (without previous life-prolonging treatment), the average survival times after non-curative treatment decision were 3.7 months (median 2, range 1–24) and 1.7 months (median 1, range 0–11), respectively. Seven (9%) patients survived over a year after the decision of non-curative treatment intent; five of these patients received life-prolonging treatment. OS after decision of treatment with non-curative intent is presented in Fig. 2. The average survival time for patients treated with non-curative intent after diagnosis of disease recurrence was 6.6 months (median 3 and range 0–30). Correspondingly, the average elapsed time after last follow-up assessment at the Departments of Otorhinolaryngology-Head and Neck Surgery or Oral and Maxillofacial Surgery to the date of death was 1.5 months (median 27 days, range 0–17 months). In the present patient series, six patients (9%) died of other causes (other cancer in five cases).

Hospital ward was the most common place of death (30 patients, 37%). Of the patients dying at a hospital ward, 37% (*n* = 11) died at a university hospital. Ten (12%) patients died on a primary health care center ward, 16 (20%) in a hospice, three (4%) in a nursing home, 13 (16%) at home, and 12 (10%) at an unknown location.

## Discussion

HNCs comprise a heterogeneous group of malignancies with site-specific characteristics for individual tumours. Oral cancer is the most common site with OTSCC being its most frequently occurring cancer type and with a steadily rising incidence in several countries [7, 8]. While the early stage OTSCC has a favourable prognosis with high cure rates, late-stage OTSCC is associated with poor OS and higher disease recurrence rates [6]. Furthermore, its treatment often results in significant morbidity. In addition to the symptomology associated with the disease itself, these patients often experience major comorbidities and complex psychosocial concerns. Life-prolonging treatment and palliative care are advocated for patients deemed incurable and for those who are non-responsive to curative treatment. The main aim remains to improve quality of life in this patient population, but, sometimes, also prolonged survival time will be achieved [1, 4]. This study was conducted to investigate the treatment concepts and patient survival for 82 patients treated for OTSCC with non-curative intent at the Helsinki University Hospital during 2005–2016, and, therefore, adds epidemiological content to the current scarce literature on the palliative care of OTSCC patients.

During the 12-year study period, a total of 1462 new tongue cancers were diagnosed in Finland according to the Finnish Cancer Registry (https://www.cancerregistry.fi). Therefore, the present series constitutes one quarter (24%) of this nation-wide cohort and, thus, forms a population-based study in Southern Finland. In our cohort, non-curative treatment intent was recommended for 7% of patients presenting with newly diagnosed primary OTSCC, most of them having Stage IV disease (83%). These findings are in accordance with the previous studies conducted in Finland [5, 9] and elsewhere [10]. For instance, Heinonen et al. [5] reported that 9% of newly diagnosed primary HNC patients at the HUS Helsinki University Hospital are referred for palliative care at presentation. In our previous nationwide study on OTSCC [6], we could additionally note that 21 (32%) out of 65 patients presenting with Stage IV disease at the first presentation were treated with non-curative intent with or without life-prolonging treatment.

A significant proportion of patients experiencing disease recurrence will eventually be recommended treatment with non-curative intent. Twenty-seven patients (23% of all patients) diagnosed with OTSCC at the Helsinki University Hospital during 2005–2009 had disease recurrence during the 5-year follow-up; 21 (78%) patients of this subgroup were eventually treated with non-curative intent [6]. This underlines the importance of an established protocol, resources, and infrastructure for palliative care, which may often have a prominent role in the treatment of patients with disease recurrence. Therefore, palliative care needs should be assessed already during the initial treatment upon the diagnosis of disease recurrence, as disease recurrence is associated with substantial symptom burden and low survival [4, 6]. In this study, while 21 (26%) patients received palliative care after curatively intended treatment of the disease recurrence (eight patients in this group also received life-prolonging treatment), in another group of 21 patients, the disease recurrence was determined incurable and consequently, a palliative care approach was implemented for most patients (three patients in this group had also previously received life-prolonging treatment).

According to studies, palliative radiotherapy is commonly advocated for patients with incurable malignant disease of the head and neck [11, 12]. In the current study, 22% of patients received radiotherapy as a palliative monotherapy and 7% with concomitant chemotherapy. A similar finding has been reported in other HNC patient series evaluated in Finland [5]. The goal of treatment in these patients is to alleviate the cancer-related devastating symptoms to improve quality of life while minimizing potential side effects, such as mucositis [12]. Therefore, radiotherapy should be given in the shortest possible time and its benefits should be judiciously pondered against the possible side effects. Moreover, in certain cases, radiotherapy can be hindered by radioresistance of the tumour, resulting in limited therapeutic gain [13]. Untreated HNC appears to be chemosensitive [14–16]. However, the efficacy of chemotherapy in recurrent disease is limited due to development of drug resistance [17]. A combination of cisplatin and 5-fluorouracil is the standard first-line treatment regimen to which new combinations are compared [18]. Studies have reported an improved survival for oral cancer patients with unresectable tumours treated with chemotherapy [19]. Chemotherapy was used only in 16% of patients in this cohort and cetuximab in combination with cisplatin and 5-fluorouracil was mostly administered. The median OS for patients treated with chemotherapy was 3 months after start of treatment. However, differences in OS among different treatment regimens could not be statistically compared due to the low number of patients in this series. Still, the short OS warrants palliative care to be initiated alongside chemotherapy.

Palliative surgery is used only in selective cases [20]. In our study, only 6% of patients underwent palliative surgery. This low figure could be explained by the sequels and complications associated with surgery of large tumours and also by the infeasibility of palliative surgery in various cases with short expectancy for survival. Almost half of the patients received only symptomatic treatment, possibly due to associated comorbidities, compromised overall physical condition, previous radiotherapy, and, in two cases, due to lack of co-operation. Treatment refusal in this patient group was rare. However, Schwam et al. [21] reported a refusal rate of 4% for postoperative radiotherapy after curative surgery. Similarly, Stavas et al. [22] reported a 3% rejection of palliative radiotherapy in lung cancer patients. To our knowledge, no studies on the prevalence of refusal of treatment with non-curative intent for HNCs have been conducted yet.

The use of PEG in patients undergoing radiotherapy can be beneficial for the patients, but carries the risk of complications. Side effects such as pain and PEG leakage, but also rare major complications, as colonic perforation due to PEG malposition and peritonitis, have been described [23, 24]. A high rate of unnecessary prophylactic PEG placement in patients with HNCs has also been reported [25]. PEG use was also not associated with improved survival, but it did reduce the need for hospitalization [26]. In the current cohort, PEG feeding was used in 65% of the patients, which is comparable to figures reported for oropharyngeal cancer patients [26]. Heinonen et al. [5] reported a 45% use of PEG in HNC patients. OTSCC is associated with functional disabilities, which can restrict food intake and affect the upper airway, plausibly justifying the relatively high percentage of PEG use in the present patient population.

The reported survival times for HNC patients treated with non-curative treatment remain short in general. Kamisetty et al. [27] reported a median survival of 4.3 months (range 2.1–8) for oral cancer patients, whereas Ledeboer et al. [10] reported a mean survival of 5.4 months and Heinonen et al. [5] reported a median survival of 3 months for HNC patients. These results are comparable to our findings with the median survival time for OTSCC patients after non-curative treatment decision being 2(<= two) months. In the present patient cohort, only seven patients (9%) survived over a year after decision of non-curative treatment. One of these patients (female, age 94) refused the proposed curatively aimed treatment for Stage II disease and, thus, received only symptomatic treatment. Two other patients (male, age 69 and 57) presenting with Stage IV disease received life-prolonging treatment without any previous treatment. The other four patients (all male, average age 65) were eventually treated with non-curative intent after disease recurrence. Still, the median survival time after diagnosis of disease recurrence was 3 months. These low figures warrant a prompt initiation of palliative care, as survival is relatively short and the complex symptoms associated with the disease can rapidly deteriorate the quality of life. The majority of patients died at the hospital and only 16% died at home. Recent studies have emphasized that the place of death influences not only the patient’s quality of life during the end-of-life period but also the mental well-being of the family members and caregivers [3, 27, 28]. Still, the majority of patients spend their final days at the hospital, which may be explained, at least partly, by the severity of the symptoms and, thus, by the warranted medical and technical expertise. In addition, the medical costs involved in home care may have a role.

Certain limitations in this retrospective study should be pointed out. One limitation is that no data were collected concerning comorbidities, which can substantially influence treatment decisions and OS. Another limitation was that we could not evaluate satisfaction for treatment and quality of life of the patients and caregivers. In addition, as mentioned previously, differences in outcome between treatment modalities could not be assessed, due to the relatively small number of patients in the cohort. Future research is warranted to examine the impact of these differences on patient survival and quality of life.

We conclude that the survival time for OTSCC patients treated primarily with non-curative intent remains very short. Still, the median treatment delay in the present study was 18–19 days, which may be considered long for this patient population.
